# The Treatment of Gonorrhœal Synovitis

**Published:** 1903-01-17

**Authors:** 


### THE TREATMENT OF GONORRHŒAL SYNOVITIS.

IN the course of an address on septic synovitis Mr. C. F. Wallis[1] deals with that form which arises from gonorrhœal infection. He says that monarticular synovitis, coming on suddenly in young people without known reason, is always suggestive of gonorrhœa. The onset of the effusion is not very painful, but it persists and does not yield to ordinary treatment. It is usually associated with a rise of temperature and accompanied by a rapid atrophy of the governing muscles. If after a week or ten days of expectant treatment—rest, strapping, salicylates, potassium iodide and mercury—the joint still remains distended, it should be opened and washed out with warm saline solution. After this has been thoroughly done the incision is closed and the limb is kept at rest until the wound is quite healed. At the end of ten to fourteen days the joint may gradually be brought into use, when massage will be found of benefit. No strong chemical lotion should be employed for washing out the joint. Sterilised saline solution is the best. The immediate results of this treatment are remarkable. "The temperature drops to normal, the pain ceases, the joint does not refill, and the patient proceeds forthwith to get well."

[1] Brit. Med. Jour., Jan. 3.

